# Imaging Characteristics of Driver Mutations in *EGFR*, *KRAS*, and *ALK* among Treatment-Naïve Patients with Advanced Lung Adenocarcinoma

**DOI:** 10.1371/journal.pone.0161081

**Published:** 2016-08-12

**Authors:** Jangchul Park, Yoshihisa Kobayashi, Kevin Y. Urayama, Hidekazu Yamaura, Yasushi Yatabe, Toyoaki Hida

**Affiliations:** 1 Department of Thoracic Oncology, Aichi Cancer Center Hospital, Nagoya, Japan; 2 Department of Respiratory Medicine, Nagoya East City Medical Center, Nagoya, Japan; 3 Department of Thoracic Surgery, Kinki University Faculty of Medicine, Osaka-Sayama, Japan; 4 Department of Human Genetics and Disease Diversity, Tokyo Medical and Dental University, Tokyo, Japan; 5 Department of Diagnostic and Interventional Radiology, Aichi Cancer Center Hospital, Nagoya, Japan; 6 Department of Pathology and Molecular Diagnostics, Aichi Cancer Center Hospital, Nagoya, Japan; Universita degli Studi di Napoli Federico II, ITALY

## Abstract

This study aimed to identify the computed tomography characteristics of treatment-naïve patients with lung adenocarcinoma and known driver mutations in *EGFR*, *KRAS*, or *ALK*. Patients with advanced lung adenocarcinoma (stage IIIB–IV) and known mutations in *EGFR*, *KRAS*, or *ALK* were assessed. The radiological findings for the main tumor and intra-thoracic status were retrospectively analyzed in each group, and the groups’ characteristics were compared. We identified 265 treatment-naïve patients with non-small-cell carcinoma, who had *EGFR* mutations (n = 159), *KRAS* mutations (n = 55), or *ALK* rearrangements (n = 51). Among the three groups, we evaluated only patients with stage IIIB–IV lung adenocarcinoma who had *EGFR* mutations (n = 126), *KRAS* mutations (n = 35), or *ALK* rearrangements (n = 47). We found that ground-glass opacity at the main tumor was significantly more common among *EGFR*-positive patients, compared to *ALK*-positive patients (*p* = 0.009). Lymphadenopathy was significantly more common among *ALK*-positive patients, compared to *EGFR*-positive patients (*p* = 0.003). Extranodal invasion was significantly more common among *ALK*-positive patients, compared to *EGFR*-positive patients and *KRAS*-positive patients (*p* = 0.001 and *p* = 0.049, respectively). Lymphangitis was significantly more common among *ALK*-positive patients, compared to *EGFR*-positive patients (*p* = 0.049). Pleural effusion was significantly less common among *KRAS*-positive patients, compared to *EGFR*-positive patients and *ALK*-positive patients (*p* = 0.046 and *p* = 0.026, respectively). Lung metastases were significantly more common among *EGFR*-positive patients, compared to *KRAS*-positive patients and *ALK*-positive patients (*p* = 0.007 and *p* = 0.04, respectively). In conclusion, *EGFR* mutations were associated with ground-glass opacity, *KRAS*-positive tumors were generally solid and less likely to metastasize to the lung and pleura, and *ALK*-positive tumors tended to present with lymphadenopathy, extranodal invasion, and lymphangitis. These mutation-specific imaging characteristics may be related to the biological differences between these cancers.

## Introduction

The recent discovery of driver mutations, such as epidermal growth factor receptor (*EGFR*) mutations and anaplastic lymphoma kinase (*ALK*) rearrangements, has dramatically changed the treatment of lung cancer [1, 2]. For example, EGFR-tyrosine kinase inhibitors, such as gefitinib, erlotinib, or afatinib, have significant benefits in patients with *EGFR* mutations [3–7]. Furthermore, one trial of crizotinib reported a remarkable response rate and prolonged progression-free survival among patients with *ALK*-positive non-small cell lung cancer (NSCLC) [8–10]. Given these clinical findings, driver mutations have become increasingly important in the era of personalized therapies, as their identification can facilitate the selection of optimally targeted drugs that provide higher response rates and fewer toxicities than cytotoxic drugs [2, 11].

We have previously demonstrated that early-stage *ALK*-positive tumors exhibit a solid pattern without ground-grass opacity (GGO) [12]. Furthermore, we observed that advanced-stage *ALK*-positive tumors exhibit specific characteristics, such as a relatively small size, lymphadenopathy, and a solid growth pattern without GGO [13]. These findings led us to hypothesize that lung cancers might exhibit distinct imaging characteristics that are dependent on the cancer’s molecular status. Therefore, we aimed to explore the potential imaging characteristics of the main tumor and intra-thoracic findings, as well as metastases and infiltration patterns, among patients with advanced-stage (stage IIIB–IV) lung adenocarcinoma and driver mutations in *EGFR*, *KRAS*, or *ALK*.

## Materials and Methods

### Cases

We retrospectively analyzed patients with treatment-naïve NSCLC who were evaluated at the Department of Thoracic Oncology of Aichi Cancer Center Hospital between July 2006 and March 2014. All patients’ medical records were reviewed to extract data regarding their clinical characteristics. This study’s design was obtained from the Ethics Committee of Aichi Cancer Center, and all patients provided written informed consent to undergo testing and treatment.

The cases were staged according to the seventh edition of The American Joint Committee on Cancer manual. Pathological specimens were obtained via transbronchial lung biopsy, endoscopic ultrasound-guided fine-needle biopsy, percutaneous core needle biopsy, or thoracentesis for pleural effusion. Each case was histologically analyzed using multiple slides from the corresponding tissue sections, which were classified according to World Health Organization’s 2004 pathology criteria. This study was approved by the Ethics Committee of the Aichi Cancer Center Hospital (Nagoya, Japan).

### Mutation analysis

Mutation analysis was performed to detect *EGFR* mutations, *KRAS* mutations, and *ALK* rearrangements. The mutation statuses of *EGFR*, *KRAS*, and *ALK* in fresh frozen tissue samples were analyzed via direct sequencing of reverse transcriptase polymerase chain reaction (RT-PCR) products. Paraffin-embedded tissues were evaluated using the Cycleave PCR technique for codons 719, 858, and 861 of *EGFR* and codon 12 of *KRAS*, and fragment analysis was used for deletions in exon 19 and insertions in exon 20 of *EGFR* [14]. Immunohistochemistry or RT-PCR was used to screen for *ALK* gene rearrangements, according to previously reported procedures [13]. If positive results were obtained using either method, the *ALK* rearrangements were confirmed using fluorescent in-situ hybridization.

### Computed tomography

Radiological findings from the initial diagnosis were retrospectively analyzed for each molecular subtype. Computed tomography (CT) was performed using 10-mm, 7-mm, 5-mm, or 2-mm of collimation, and all CT scans were reviewed to evaluate the main tumor’s characteristics and intra-thoracic status. The main tumor was evaluated for the presence of a solid appearance (>50% solid components), partially solid appearance (≤50% solid components), consolidation, GGO, air bronchograms, and size (>3 cm or ≤3 cm). Intra-thoracic findings were evaluated for the presence of adenopathy (defined as hilar, mediastinal, subclavicular, or axillary nodes with a short-axis dimension of ≥1 cm), extranodal invasion of the lymph nodes, lymphangitis, pleural effusion, pericardial effusion, and lung metastases. Consolidation on the CT image was defined as increased density of the lung parenchyma with obscuring of the pulmonary vessels, and GGO was defined as a hazy increase in attenuation that did not obscure the normal lung markings. Air bronchograms on the CT image were defined as air-filled bronchi that appeared as radiolucent branching bands within pulmonary densities. Extranodal invasion was defined as invasion of the adjacent tissue. All radiological terms were defined based on the Fleischner Society’s glossary terms [15]. The largest diameter measurements in all cases were calculated manually using the picture archiving and communication system’s electronic measurement tool. One radiologist (HY) and two chest physicians (JP and TH) reviewed the CT scans and reached their conclusions via consensus.

### Statistical analysis

The characteristics of each molecular subtype were compared to the other subtypes (e.g., *EGFR*-positive patients were compared to *KRAS*-positive patients and *ALK*-positive patients). Furthermore, *EGFR*-positive patients were subdivided into three groups (exon 19 deletions, exon 21 L858R point mutations, and other mutations), and these groups were also compared. Patient characteristics were compared using the Wilcoxon/Kruskal–Wallis test or the χ^2^ test, as appropriate. For the radiological analyses, we used Fisher’s exact test or the χ^2^ test, as appropriate. Differences with a *p*-value of <0.05 were considered statistically significant, and all analyses were performed using JMP software (version 10.0; SAS Institute Inc., Cary, NC, USA).

## Results

### Patients’ characteristics

We identified 265 patients with driver mutations in *EGFR* (n = 159, 60%), *KRAS* (n = 55, 20.8%), and *ALK* (n = 51, 19.2%) (Table 1). However, one case with mutations in both *EGFR* and *KRAS* was excluded. The *ALK*-positive patients were generally diagnosed at a younger age (median age: 51 years), whereas patients with the other molecular subtypes were diagnosed at similar ages. Patients with *KRAS* mutations were significantly more likely to be male, compared to patients with *EGFR* mutations (*p* < 0.001) or *ALK* mutations (*p* < 0.001). There were also significant differences in the proportions of men and women in the subtype groups (*p* < 0.001). The majority of patients were diagnosed at stage IIIB–IV (stage I–IIIA vs. IIIB–IV *p* = 0.02). The distributions of mutations in *EGFR* were 73 (45.9%) exon 19 deletions, 64 (40.2%) exon 21 L858R point mutations, and 20 (13.9%) other mutations.

**Table 1:** Patients’ characteristics.

Characteristics	Genetic alteration				*p* value		
	Total	*EGFR+*	*KRAS*+	*ALK*+	*EGFR*+ vs. *KRAS*+	*EGFR*+ vs. *ALK*+	*KRAS*+ vs. *ALK*+
N	265	159 (60)	55 (20.8)	51 (19.2)			
Median age, years (range)	62.1 (29–89)	65 (30–89)	64 (36–84)	51 (29–80)	0.445	**<0.001**	**<0.001**
Sex					**<0.001**	0.317	**<0.001**
Male	143 (54)	55 (34.6)	45 (81.8)	22 (43.1)			
Female	122 (46)	104 (65.4)	10 (18.2)	29 (56.9)			
Histology					**<0.001**	0.734	**0.041**
Adenocarcinoma	246 (92.8)	154 (96.9)	42 (76.3)	50 (98)			
Adenosquamous	1 (0.4)	0 (0)	1 (1.8)	0 (0)			
Squamous	7 (2.6)	2 (1.3)	5 (9.1)	0 (0)			
Large cell	4 (1.5)	1 (0.6)	3 (5.5)	0 (0)			
LCNEC	1 (0.4)	1 (0.6)	0 (0)	0 (0)			
NOS	5 (1.9)	1 (0.6)	3 (5.5)	1 (2)			
Sarcomatoid	1 (0.4)	0 (0)	1 (1.8)	0 (0)			
Stage							
I	8 (3)	7 (4.4)	1 (1.8)	0 (0)	0.831	**0.027**	0.054
II	6 (2.3)	5 (3.1)	1 (1.8)	0 (0)			
IIIA	30 (11.3)	19 (12)	8 (14.6)	3 (5.9)			
IIIB	24 (9.1)	7 (4.4)	11 (20)	6 (11.7)			
IV	197 (74.3)	121 (76.1)	34 (61.8)	42 (82.4)			
*EGFR* mutation typ*e*							
19DEL		73 (45.9)	-	-			
L858R		64 (40.2)	-	-			
Other		22 (13.9)	-	-			

EGFR, epidermal growth factor receptor; KRAS, Kirsten rat sarcoma; ALK, anaplastic lymphoma kinase; LCNEC, large cell neuroendocrine carcinoma; NOS, not otherwise specified; Sarcomatoid, sarcomatoid carcinoma

### Main tumor findings

**Fig 1:**
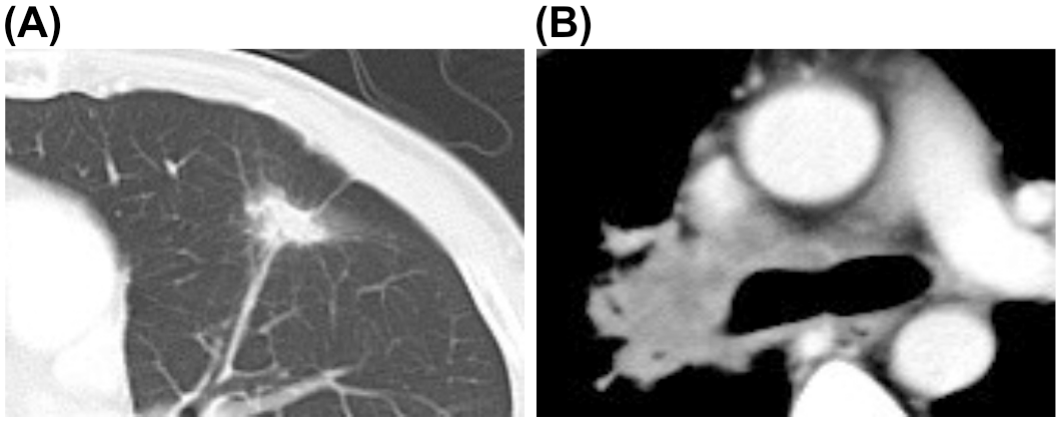
Computed tomography images of a lung adenocarcinoma showing ground-glass opacity with solid components. (A) Thoracic computed tomography reveals ground-glass opacity with solid components. (B) Thoracic computed tomography reveals extranodal invasion of the lymph nodes.

Among the three groups, we evaluated only patients with stage IIIB–IV lung adenocarcinoma who had *EGFR* mutations (n = 126), *KRAS* mutations (n = 35), or *ALK* rearrangements (n = 47) to avoid the influence of confounding factors such as tissue type and staging. The radiological findings regarding the main tumor are shown in Table 2. The majority of the patients exhibited solid tumors, rather than partially solid tumors. However, significant differences were observed in the distributions of having any GGO components (*p* = 0.014), and GGO components were significantly more common among *EGFR*-positive patients, compared to *ALK*-positive patients (*p* = 0.009). Air bronchograms were significantly more common among *EGFR*-positive patients, compared to *KRAS*-positive patients (*p* = 0.04). *ALK*-positive patients were more likely to have a small tumor (≤ 3 cm), compared to *EGFR*-positive patients, although this difference was not statistically significant (*p* = 0.06). A representative example of the CT findings of GGO with solid components is shown in Fig 1A.

**Table 2:** Imaging characteristics of the main tumor in stage IIIB–IV patients with lung adenocarcinoma.

Characteristics	Genetic alteration			*p* value		
	*EGFR+*	*KRAS*+	*ALK*+	*EGFR*+ vs. *KRAS*+	*EGFR*+ vs. *ALK*+	*KRAS*+ vs. *ALK*+
N	126	35	47			
Solid	117 (92.9)	34 (97.1)	42 (89.4)	0.353	0.453	0.181
Part solid	4 (3.2)	0 (0)	0 (0)	0.286	0.217	1
Any GGO	21 (16.7)	2 (5.7)	1 (2.1)	0.101	**0.009**	0.392
Consolidation	9 (7.1)	1 (2.9)	5 (10.6)	0.353	0.453	0.181
Air bronchograms	31 (24.6)	3 (8.6)	7 (14.9)	**0.04**	0.17	0.387
Size > 3cm	89 (70.6)	24 (68.6)	26 (55.3)	0.813	0.06	0.224

EGFR, epidermal growth factor receptor; KRAS, Kirsten rat sarcoma; ALK, anaplastic lymphoma kinase; GGO, ground-grass opacity.

### Intra-thoracic findings

**Fig 2:**
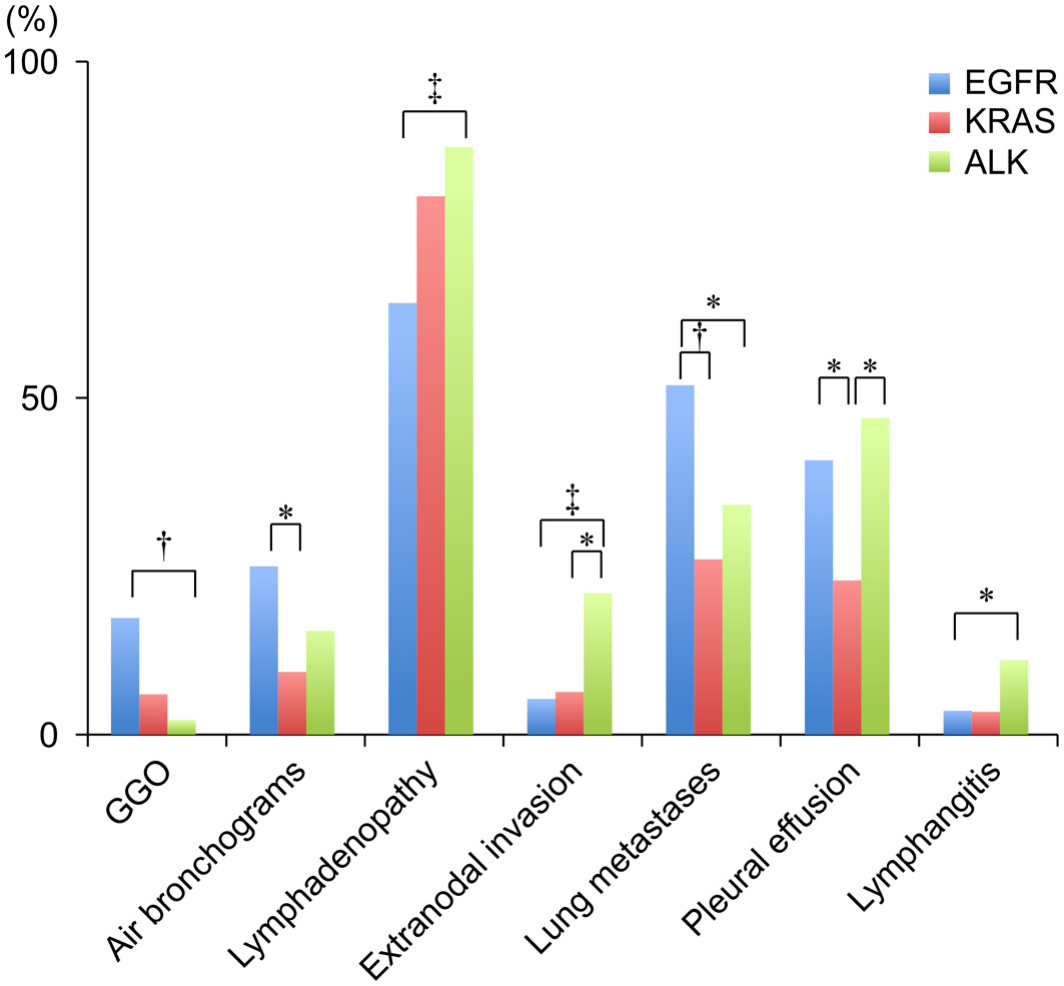
The relative distributions of the main tumor and intra-thoracic characteristics among stage IIIB–IV patients with lung adenocarcinoma. *P < 0.05; † *P* < 0.01; ‡ *P* < 0.005.

Significant differences were observed between the three molecular subtypes in their distributions of lymphadenopathy, extranodal invasion, and lung metastases (*p* = 0.006, *p* = 0.002, and *p* = 0.008, respectively). The radiological characteristics of intra-thoracic findings are shown in Table 3. *KRAS*-positive patients and *ALK*-positive were more likely to have lymphadenopathy, compared to *EGFR*-positive patients (*p* = 0.079 and *p* = 0.003, respectively). *ALK*-positive patients were significantly more likely to have extranodal invasion, compared to *EGFR*-positive patients or *KRAS*-positive patients (*p* = 0.001 and *p* = 0.049, respectively). Representative CT finding of extranodal invasion are shown in Fig 1B. *EGFR*-positive patients were significantly more likely to have lung metastases, compared to *KRAS*-positive patients or *ALK*-positive patients (*p* = 0.007 and *p* = 0.04, respectively). Pleural effusion was significantly less common among *KRAS*-positive patients, compared to *EGFR*-positive patients or *ALK*-positive patients (*p* = 0.046 and *p* = 0.026, respectively). *ALK*-positive patients were significantly more likely to have more lymphangitis, compared to *EGFR*-positive patients (*p* = 0.049). The relative distributions of the various characteristics among the different molecular subtypes are shown in Fig 2.

**Table 3:** Imaging characteristics of the intra-thoracic findings in stage IIIB–IV patients with lung adenocarcinoma.

Characteristics	Genetic alteration			*p* value		
	*EGFR+*	*KRAS*+	*ALK*+	*EGFR*+ vs. *KRAS*+	*EGFR*+ vs. *ALK*+	*KRAS*+ vs. *ALK*+
N	126	35	47			
Lymphadenopathy	81 (64.3)	28 (80)	41 (87.2)	0.079	**0.003**	0.375
Extranodal invasion	6 (4.8)	2 (5.7)	10 (21.3)	0.685	**0.001**	**0.049**
Lung metastases	65 (51.6)	9 (25.7)	16 (34)	**0.007**	**0.04**	0.418
Pleural effusion	52 (41.3)	8 (22.9)	22 (46.8)	**0.046**	0.513	**0.026**
Pericardial effusion	4 (3.2)	2 (5.7)	3 (6.4)	0.483	0.341	0.9
Lymphangitis	4 (3.2)	1 (2.9)	5 (10.6)	0.924	**0.049**	0.181

EGFR, epidermal growth factor receptor; KRAS, Kirsten rat sarcoma; ALK, anaplastic lymphoma kinase.

### Radiological findings according to EGFR mutation subtype

We also analyzed the CT characteristics of the various *EGFR* mutation subtypes. However, there were no significant differences in the characteristics of the patients with exon 19 in-frame deletions, exon 21 L858R point mutations, and other mutations (S1 Table).

## Discussion

In this study, we found that *EGFR* mutations were associated with GGO, *KRAS* mutations were associated with solid tumors that had low tendencies to metastasize to the lung and pleura, and *ALK* rearrangements were associated with lymph node involvement and lymphangitis. To the best of our knowledge, this is the first study to evaluate the intra-thoracic radiological findings of advanced-stage lung adenocarcinoma with mutations in three common oncogenes: *EGFR*, *KRAS*, and *ALK*.

Our findings indicate that advanced *ALK*-positive lung adenocarcinoma was significantly associated with the presence of lymphadenopathy, lymph nodes with extranodal invasion, lymphangitis, and pleural effusion, and that these tumors were less likely to exhibit GGO components. We have also previously reported that the main tumor in early-stage *ALK*-positive lung adenocarcinoma is typically solid, with few GGO components and a low tumor disappearance rate [12]. Although we did not evaluate these specific characteristics in the present study, our results confirm that *ALK*-positive lung adenocarcinomas are typically solid, even at the advanced stage, which is likely due to their distinct pathological states [16, 17].

Our findings regarding the metastatic nature of *ALK* rearrangements are supported by those of other studies. For example, Choi et al. reported that *ALK*-positive lung cancers exhibited a higher maximum standardized uptake value, and more common metastasis to lymph nodes and other organs, compared to *EGFR*-positive and wild-type lung cancers [18]. Furthermore, Halpenny et al. reported that *ALK*-positive lung adenocarcinomas are more likely to exhibit multifocal thoracic lymphadenopathy, compared to *EGFR*-positive lung adenocarcinomas, although they only included a relatively small number of *ALK*-positive patients (8 cases of stage IIIB and 18 cases of stage IV) [19]. Moreover, Doebele et al. reported that *ALK*-positive patients exhibited higher incidences of pericardial, pleural, and liver metastases [20]. Given that *ALK*-positive lung cancer involves the lymph nodes, there are several clinical treatment strategies (e.g., lymph node radiation after resection of the *ALK*-positive lung cancer). In addition, these characteristics of *ALK*-positive lung cancer could be useful in difficult-to-diagnose cases (e.g., IHC-positive and FISH-negative or IHC-negative and FISH positive cases) [21, 22] However, these strategies are not supported by any reports or clinical trials, and to our best knowledge, little is known regarding the recurrence patterns for the various molecular subtypes. Therefore, future studies should examine the recurrence patterns for specific molecular subtypes, as this issue was outside the scope of the present study. Nevertheless, we confirmed that *ALK*-positive lung adenocarcinoma is typically solid, aggressive, and more likely to involve the local lymphatic system, compared to *EGFR*-positive lung cancer.

We also found that *ALK*-positive patients tended to have smaller tumors (≤ 3 cm) compared to those in *EGFR*-positive patients, suggesting that genomic state could influence the tumor size. Although this was not statistically significant (*p* = 0.06), this could be due to the low sample size. Further investigations of the relationship between genetic alterations and tumor sizes are warranted.

Our analyses also revealed that *EGFR* mutations were significantly associated with GGO components and air bronchograms. Other studies have reported that *EGFR* mutations were correlated with GGO components [23, 24, 25], although those studies typically examined early-stage cancers. Therefore, the clinical implication of GGO in advanced-stage lung cancer remains undetermined. However, Hsu et al. reported that advanced lung adenocarcinomas with wild-type *EGFR* were significantly associated with a larger tumor size and irregular shape, and that tumors with exon 19 deletions were associated with air bronchograms [26]. These findings are consistent with our results, although we did not observe any significant differences between the different *EGFR* mutation subtypes, which may be related to the relatively small number of patients with each mutation. Therefore, it may be difficult to draw any definitive conclusions regarding the main tumor’s mutation-specific characteristics in advanced-stage *EGFR*-positive lung cancer, although our findings indicate that *EGFR*-positive tumors exhibit unique characteristics, compared to the other molecular subtypes.

The present study also revealed that *EGFR* mutations in advanced lung carcinoma were more frequently associated with intra-thoracic metastases to the lung and pleura, and the absence of lymphadenopathy and extranodal invasion, compared to the *KRAS* and *ALK* mutations. Other studies have reported that distant metastases are observed in patients with *EGFR* mutations, which is consistent with this molecular subtype being associated with hematogenous metastasis [20, 27]. Furthermore, Shin et al. reported that *EGFR* mutations were associated with the presence and number of brain metastases among patients with pulmonary adenocarcinoma at the initial diagnosis [28]. In contrast, several studies have reported that *EGFR* mutations are associated with lower nodal stages [28, 29]. Therefore, our results suggests that *EGFR*-positive lung cancers exhibit more GGO components, less lymph node invasion, and a higher tendency to metastasize to other organs, compared to the *KRAS*- and *ALK*-positive subtypes.

Examining the correlations between specific imaging phenotypes and genomic findings is an emerging research topic. This field, which is called “radiogenomics,” uses novel methods to examine the associations between radiological imaging phenotypes and gene expression patterns, and is anticipated to address the complex topics of cancer progression, metastasis, therapy resistance, and recurrence [30, 31]. Thus, imaging analyses are increasingly important for clinicians to implement personalized medicine that tailors the patient’s treatment based on their molecular characteristics. The present study has several limitations. First, the retrospective single-center design is associated with various potential biases. Second, not all patient examinations were performed using the same protocol. Third, the slice thicknesses for the CT examinations might have influenced the radiological findings and our calculation of the main tumor’s size. Fourth, we did not evaluate the molecular variants among *KRAS*-positive and *ALK*-positive patients, which might have provided more detailed information. Therefore, larger studies are needed to examine the precise natures and characteristics of these driver mutations. Fifth, we only evaluated advanced-stage lung cancers, not early-stage lung cancers. Sixth, although *ALK*-positive patients were more likely to have a small tumor compared to *EGFR*-positive patients, this may be because of indolent nature of *EGFR*-positive tumors.

## Conclusions

Patients with *ALK*-positive lung adenocarcinoma tended to present with lymphadenopathy, extranodal invasion, and lymphangitis. In contrast, patients with *EGFR*-positive lung adenocarcinoma tended to present with GGO, and patients with *KRAS*-positive lung adenocarcinoma tended to present with a solid tumor that had limited tendency to metastasize to the lung and pleura. These findings indicate that lung cancers have distinct imaging characteristics that are determined by their specific molecular status. Therefore, this information may help clinicians define appropriate follow-up and treatment strategies for patients with NSCLC, based on their molecular status.

## Supporting Information

S1 Table Imaging characteristics according to *EGFR* mutation subtypes in stage IIIB–IV patients with lung adenocarcinoma. (DOCX)
