# Supplementary material for: Imaging Characteristics of Driver Mutations in EGFR, KRAS, and ALK among Treatment-Naïve Patients with Advanced Lung Adenocarcinoma
Source: PLoS One. 2016 Aug 12;11(8):e0161081. doi: 10.1371/journal.pone.0161081 (PMC4982673; doi:10.1371/journal.pone.0161081)
Supplement: S1 Table — (DOCX) [file pone.0161081.s001.docx]

**Imaging Characteristics of Driver Mutations in EGFR, KRAS, and ALK among Treatment-naïve Patients with Advanced Lung Adenocarcinoma**

Jangchul Park^1,2^, Yoshihisa Kobayashi^3^, Kevin Y. Urayama^4^, Hidekazu Yamaura^5^, Yasushi Yatabe^6^, and Toyoaki Hida1^*^

**S1 Table. Imaging characteristics according to *EGFR* mutation subtypes in stage IIIB–IV patients with lung adenocarcinoma**

| Characteristics | Genetic alteration | | |  | *P* value | | |
| --- | --- | --- | --- | --- | --- | --- | --- |
|  | *19DEL* | *L858R* | *Other* |  | *19DEL* vs. *L858R* | *19DEL* vs. *Other* | *L858R* vs. *Other* |
| N | 60 | 51 | 13 |  |  |  |  |
| Solid | 54 (90) | 48 (94.1) | 13 (100) |  | 0.428 | 0.234 | 0.37 |
| Part solid | 3 (5) | 1 (2) | 0 (0) |  | 0.392 | 0.41 | 0.611 |
| Any GGO | 12 (20) | 8 (15.7) | 1 (7.7) |  | 0.556 | 0.293 | 0.459 |
| Consolidation | 4 (6.7) | 4 (7.8) | 1 (7.7) |  | 0.811 | 0.894 | 0.986 |
| Air bronchograms | 14 (23.3) | 13 (25.5) | 3 (23.1) |  | 0.792 | 0.984 | 0.858 |
| Size > 3cm | 44 (73.3) | 33 (64.7) | 10 (76 .9) |  | 0.326 | 0.789 | 0.402 |
| Lymphadenopahy | 36 (60) | 34 (66.7) | 10 (76.9) |  | 0.468 | 0.252 | 0.476 |
| Extranodal invasion | 1 (1.7) | 3 (5.9) | 1 (7.7) |  | 0.235 | 0.228 | 0.81 |
| Lung metastases | 34 (56.7) | 25 (49) | 6 (46.2) |  | 0.421 | 0.49 | 0.854 |
| Pleural effusion | 26 (43.3) | 22 (43.1) | 4 (30.8) |  | 0.983 | 0.404 | 0.418 |
| Pericardial effusion | 2 (3.3) | 2 (3.9) | 0 (0) |  | 0.868 | 0.505 | 0.468 |
| Lymphangitis | 2 (3.3) | 2 (3.9) | 0 (0) |  | 0.868 | 0.505 | 0.468 |

EGFR, epidermal growth factor receptor; 19DEL, *EGFR* exon 19 in-frame deletion; L858R, *EGFR* exon 21 L858R point mutation; GGO, ground-grass opacity.
